# Quantitative Phosphoproteomics Reveals a Role for Collapsin Response Mediator Protein 2 in PDGF-Induced Cell Migration

**DOI:** 10.1038/s41598-017-04015-x

**Published:** 2017-06-21

**Authors:** Adil R. Sarhan, Justyna Szyroka, Shabana Begum, Michael G. Tomlinson, Neil A. Hotchin, John K. Heath, Debbie L. Cunningham

**Affiliations:** 1 School of Biosciences, University of Birmingham, Edgbaston, Birmingham B15 2TT United Kingdom; 2 MRC Protein Phosphorylation and Ubiquitylation Unit, College of Life Sciences, University of Dundee, Dow Street, Dundee, DD1 5EH United Kingdom

## Abstract

The Platelet Derived Growth Factor (PDGF) family of ligands have well established functions in the induction of cell proliferation and migration during development, tissue homeostasis and interactions between tumours and stroma. However, the mechanisms by which these actions are executed are incompletely understood. Here we report a differential phosphoproteomics study, using a SILAC approach, of PDGF-stimulated mouse embryonic fibroblasts (MEFs). 116 phospho-sites were identified as up-regulated and 45 down-regulated in response to PDGF stimulation. These encompass proteins involved in cell adhesion, cytoskeleton regulation and vesicle-mediated transport, significantly expanding the range of proteins implicated in PDGF signalling pathways. Included in the down-regulated class was the microtubule bundling protein Collapsin Response Mediator Protein 2 (CRMP2). In response to stimulation with PDGF, CRMP2 was dephosphorylated on Thr514, an event known to increase CRMP2 activity. This was reversed in the presence of micromolar concentrations of the protein phosphatase inhibitor okadaic acid, implicating PDGF-induced activation of protein phosphatase 1 (PP1) in CRMP2 regulation. Depletion of CRMP2 resulted in impairment of PDGF-mediated cell migration in an *in vitro* wound healing assay. These results show that CRMP2 is required for PDGF-directed cell migration *in vitro*.

## Introduction

Platelet Derived Growth Factor (PDGF) was first identified in platelet extracts as a potent mitogen for fibroblasts and endothelial cells^1^. The PDGF family of ligands comprises four related gene products: PDGFA, PDGFB, PDGFC and PDGFD which can form both homodimers and heterodimers providing a repertoire of PDGF isoforms^2^. The PDGF family of ligands elicit their biological effects by interacting with two structurally related high affinity receptors of the tyrosine kinase family: PDGFR alpha and PDGFR beta^3^. PDGF-AA, AB, BB and CC induce PDGFR alpha homo-dimerisation; PDGF-BB and DD induce formation of PDGFR beta homodimers, and PDGF-AB, BB, CC and DD induce formation of PDGFR alpha/PDGFR beta heterodimers^2^. PDGF-BB homodimers are therefore universal PDGFR ligands capable of eliciting the full repertoire of PDGFR-mediated biological responses.

PDGF receptors are expressed by cells of mesenchymal origin and the cognate PDGF ligands are expressed by overlying epithelial cells indicative of paracrine signalling from epithelium to the underlying mesenchyme. For example, in the lung, PDGFR alpha is expressed in the lung mesenchyme and PDGFA is expressed in the epithelium^4^. This pattern is seen in multiple organs and tissues such as gut, kidney, gonads, somites and nervous system^5^. Extensive genetic studies of PDGF and PDGFR function in the mouse confirms that PDGF signalling from epithelial cells via PDGFRs is absolutely required for the expansion and migration of specific mesenchymal cell populations^6^. For example, in the kidney, PDGF signalling is required both for the multiplication of mesangial cell precursors and their migration into the glomerular space^5^. In the lung, PDGF is required for the expansion of myofibroblasts and their migration along the alveolar epithelium during lung morphogenesis^7^. In the central nervous system (CNS) oligodendrocyte precursor (O2A) cells require PDGF signalling both for population expansion and migration through the spinal cord^8^.

PDGF signalling also has significant functions in adult tissue homeostasis and regeneration, acting on cells of mesenchymal origin. PDGF is an established mitogen and chemoattractant for mesenchymal derived cells *in vitro* ^9^ and has been studied for its ability to promote wound healing through promoting the migration, proliferation and extracellular matrix deposition by fibroblasts^10^. Ectopic expression of PDGFB in the kidney glomerulus induces expansion of bone marrow derived mesenchymal cells to form a severe mesanagial glomerulopathy and fibrosis^11^. PDGF-BB is a mitogen for vascular smooth muscle cells and activation of PDGF signalling is required for pulmonary arterial hypertension (PAH) and pulmonary arterial remodelling^12^.

The PDGF signalling axis is also implicated in regulation of tumour/stromal cell interactions^13, 14^. PDGF-BB produced by tumours of epithelial origin acts upon adjacent connective tissue cells, pericytes and vascular smooth muscle cells to regulate tumour angiogenesis and tumour-associated fibrosis^15^. PDGF-activated perivascular cells also promote metastasis via tumour-associated macrophages and act on tumour cells that have undergone epithelial-mesenchymal transition (EMT) to promote migration through the extracellular matrix^16, 17^.

Despite the biological significance of these activities, little is known about the downstream intracellular pathways that mediate these responses beyond canonical RTK signalling pathways such as PLC, AKT and MAPK/ERK^2^. To gain further insight into how PDGFs mediate their biological effects we analysed a differential phosphoproteomics dataset of PDGF-stimulated mouse embryo fibroblasts (MEFs)^18^. This shows that, in MEFs, PDGFR activation elicits a broad range of phosphorylation events mediated by downstream Ser/Thr kinases which impact on mediators of cytoskeletal function and cell motility. In particular, we show that Collapsin response mediator protein-2 (CRMP2) is dephosphorylated in response to PDGF and is required for PDGF-induced cell migration. CRMP2 (also known as dihydropyrimidinase-like 2) is highly expressed in the nervous system and mediates cellular responses downstream of ephrins, neurotrophins and semaphorins, such as neuronal polarity, growth and axonal guidance^19, 20^.

Collectively these results significantly expand the range of proteins and phosphorylation events implicated in PDGF signalling pathways and reveal potential new targets for therapeutic intervention in PDGF signalling. They also reveal a role for CRMP2 in PDGF-induced cell migration which has broad implications for tissue repair and tumour development.

## Results

### PDGF-induced changes in the phosphoproteome

**Figure 1:**
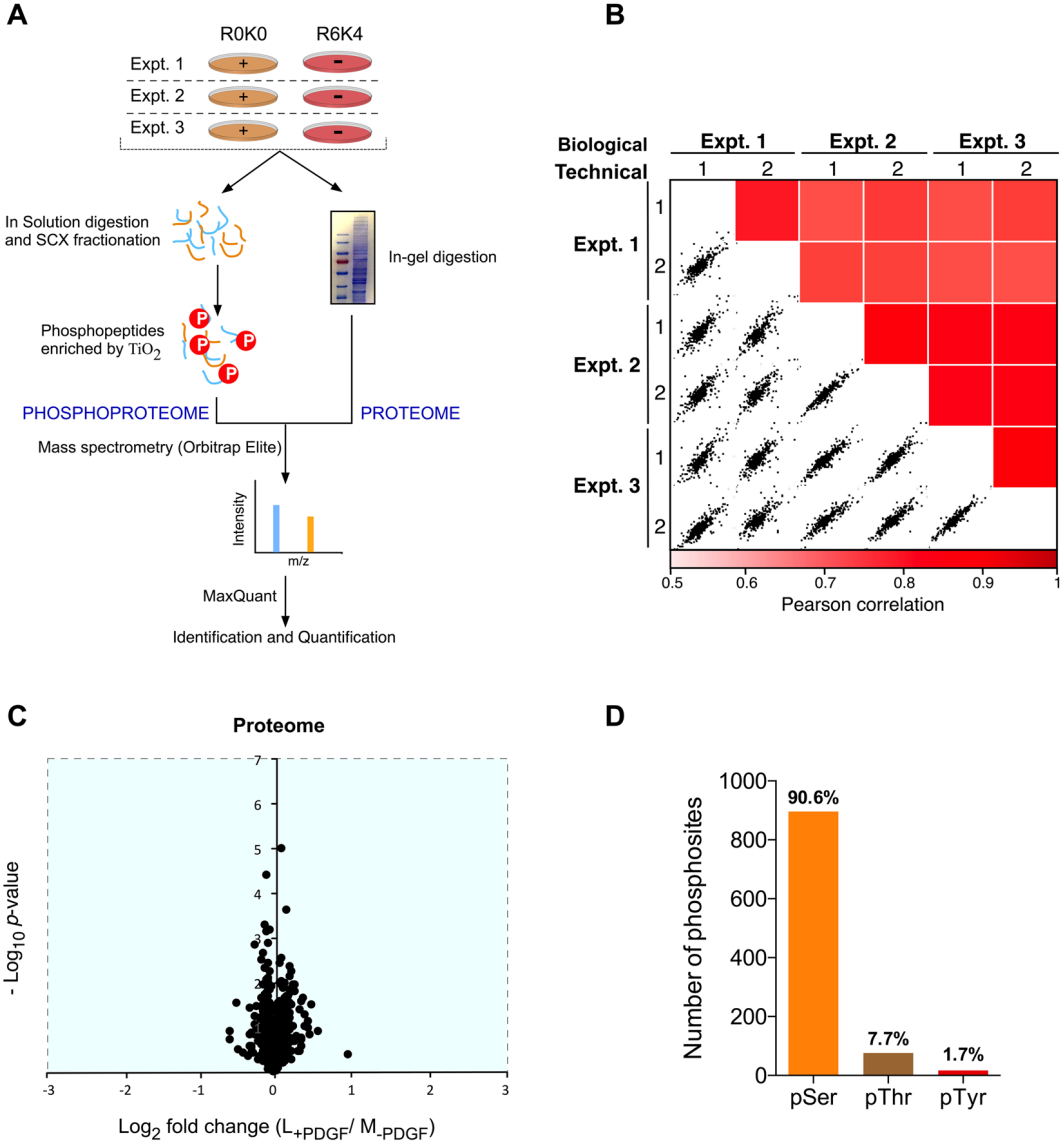
Global quantitative proteomics and phosphoproteomics of PDGF signalling in mouse embryo fibroblasts (MEFs). (**A**) Schematic overview of experimental design. SILAC labelled MEFs were stimulated with PDGF for 7 min (+) or left unstimulated (−). (**B**) Heat map of Pearson correlation coefficients and multi scatter plots demonstrating reproducibility between the phosphoproteome of biological and technical replicates. (**C**) Volcano plots showing the ratio (log2 fold change) and significance (−log10 Benjamini-Hochberg adjusted *p*-value) of the proteome dataset. (**D**) Numbers of serine, threonine and tyrosine phosphorylation sites identified within our PDGF-regulated phosphoproteome dataset.

**Figure 2:**
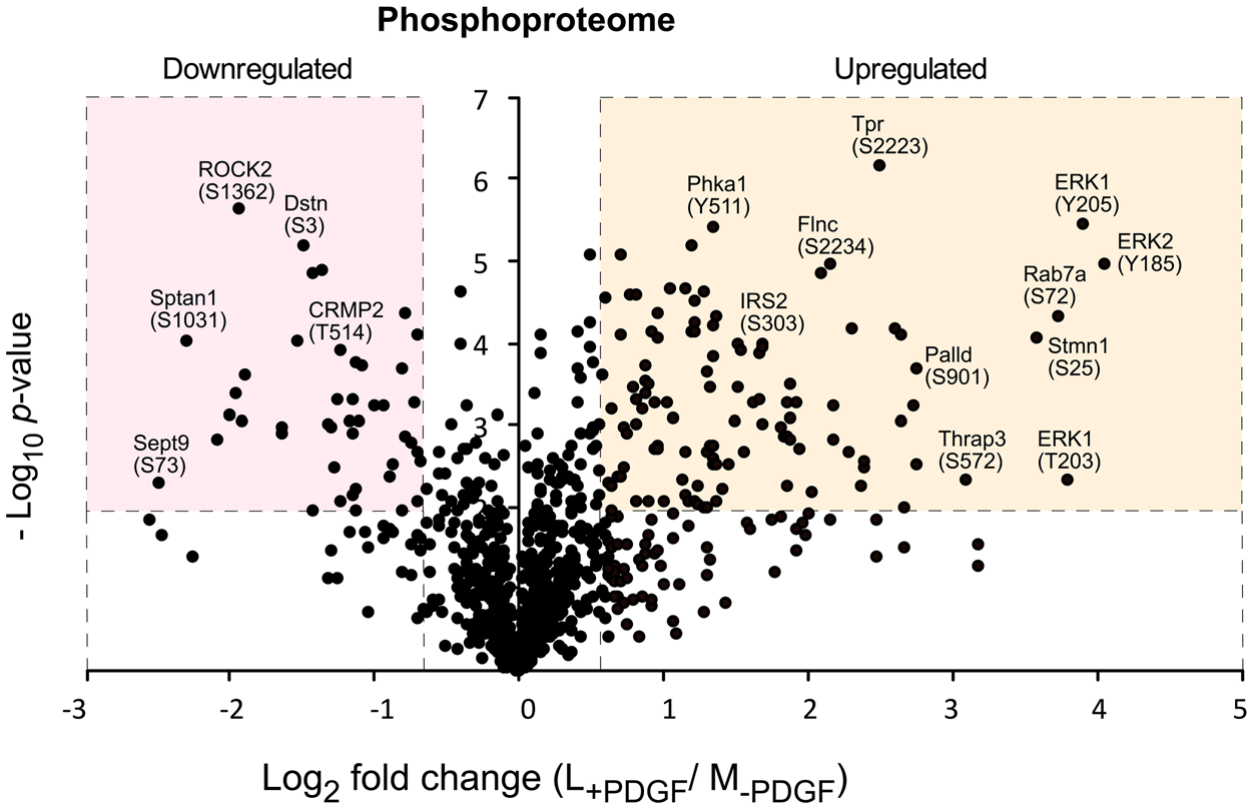
Volcano plots showing the magnitude and significance of differential phosphopeptide abundance in PDGF-stimulated versus unstimulated cells. Phosphopeptides are deemed significantly up- or down-regulated if they have an adjusted p value < 0.05 and exhibit a >1.5 fold-change in abundance.

**Figure 3:**
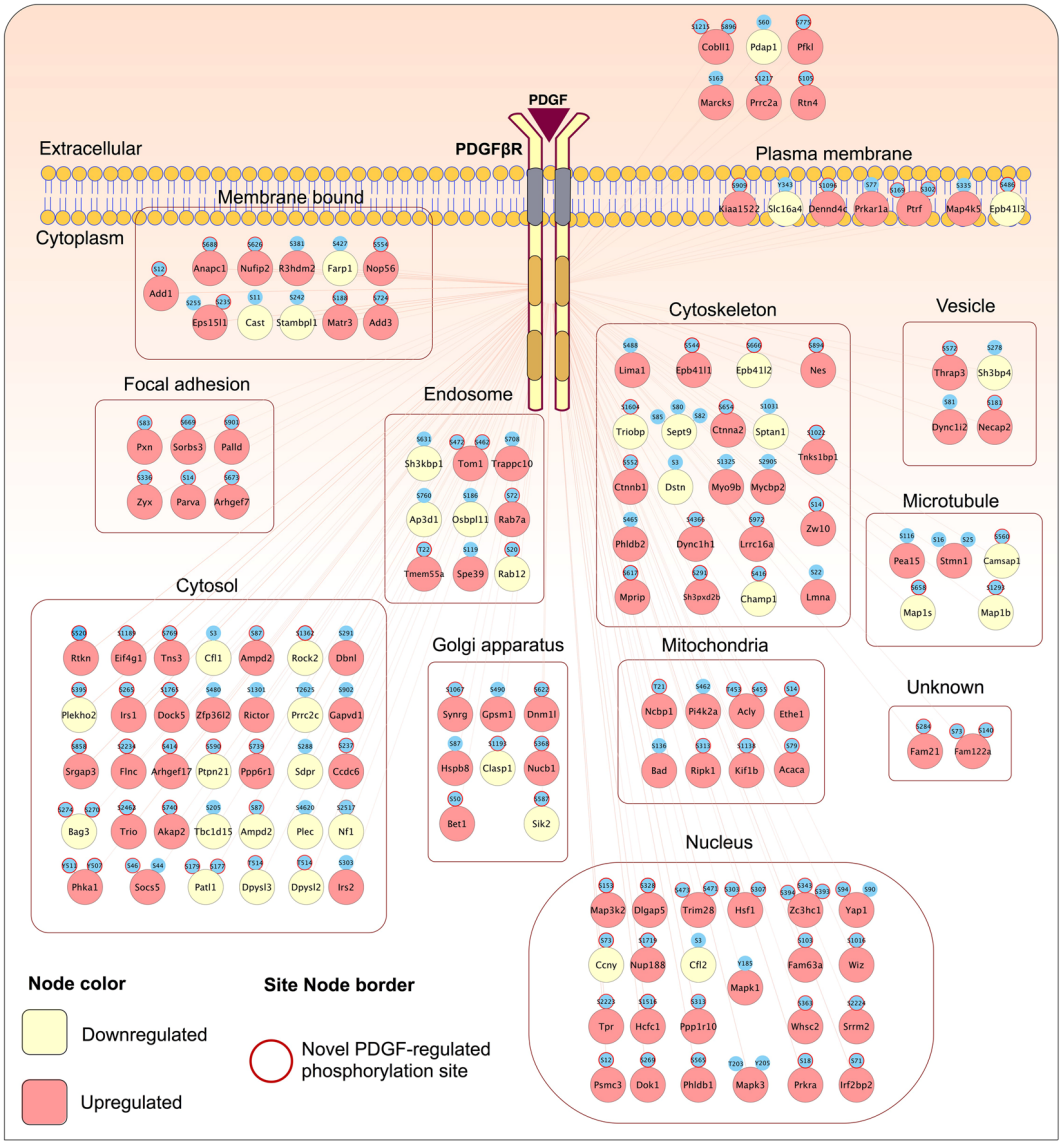
PDGF-regulated proteins and phosphosites. Proteins containing PDGF-regulated phosphosites were clustered according to their subcellular localisations as listed in the COMPARTMENTS database. PDGF-regulated phosphosites on each protein are shown in blue and those that are novel PDGF-regulated sites are bordered in red.

We have recently published quantitative SILAC proteomics data identifying changes in the phosphoproteome and proteome of mouse embryonic fibroblasts (MEFs) due to the absence of the intracellular phosphatase domain of Leukocyte Common Antigen-related (LAR) protein tyrosine phosphatase (PRIDE Accession PXD002545^18, 21^). As part of our analysis of LAR function WT MEFs were stimulated with PDGF, and here we have reanalysed this part of the dataset to identify novel PDGF-dependent phosphorylation events. Full details of the experimental setup and procedures used to obtain the phosphoproteomics and proteomics datasets have been previously described^18^. Methodology relevant to the PDGF-dependent dataset are summarised in Fig. 1A. The Pearson’s correlation coefficient for the phosphopeptide ratios measured across the three biological replicates ranged from 0.77 to 0.93 indicating good biological reproducibility (Fig. 1B). Within the proteomic dataset, a total of 703 proteins were identified in two or more biological replicates. No significant differences in protein abundance were observed between unstimulated and PDGF stimulated (7 min) cells (Fig. 1C). In the phosphoproteome dataset, 989 phosphosites from 611 proteins were detected with high localization scores (localization probability >0.75; score difference >5) in three or more experimental replicates (Supplementary Table S1). Within the phosphoproteome dataset, we identified 896 (90.6%) serine, 76 (7.7%) threonine and 17 (1.7%) tyrosine phosphorylation sites (Fig. 1D). These results are an accurate reflection of the relative abundance of serine:threonine:tyrosine phosphorylation events in the cell, previously reported to be 88:11:1, and are comparable to those detected downstream of other receptor tyrosine kinases^22, 23^. Being a tyrosine kinase, activation of PDGFR will initiate cell signalling via tyrosine phosphorylation. PDGF-induced tyrosine phosphorylation events have been previously studied using enrichment strategies to increase the relative abundance of tyrosine phosphorylated peptides^24, 25^. As our aim was to gain an understanding of the global signalling landscape regulated by PDGF our enrichment strategy was not selective for either serine, threonine or tyrosine, enabling novel insight into the breadth of biological processes regulated by PDGF. From the 989 phosphopeptides identified, 161 showed a significant change in phosphorylation status upon PDGF stimulation (p < 0.05; >1.5-fold change) (Fig. 2; Supplementary Table S2). A total of 116 phosphosites were upregulated and 45 downregulated upon PDGF treatment. Cellular compartment analysis revealed that these PDGF-regulated phosphosites are present on proteins localised throughout the cell, indicating the diversity of PDGF signalling (Fig. 3).

Within this dataset we identified proteins that have previously been shown to be involved in PDGF signalling. For example, the ERK1/2 MAP kinases have been extensively studied and shown to be hyperphosphorylated in response to growth factors such as PDGF^26–28^. In agreement with these studies, we found increased phosphorylation of ERK1 on Tyr205 (L_+PDGF 7min/_M_−PDGF_; 15.10) and Thr203 (L_+PDGF_/M_−PDGF_; 14.13), and ERK2 on Tyr185 (L_+PDGF_/M_−PDGF_; 16.77). The phosphorylation of these sites on ERK1/2 is essential for kinase activity. Phosphorylated ERK1/2 mediates diverse physiological functions such as cell growth, survival, cell adhesion, migration and differentiation^26–28^. We have also identified an increase in the phosphorylation of Bcl2-associated agonist of cell death (BAD) at Ser136 upon PDGF stimulation (Supplementary Table S2). This site is a target for Akt downstream of PDGF and phosphorylation of Ser136 on BAD leads to enhanced cell survival^29^.

### Novel PDGF-regulated phosphosites

From 161 phosphosites, we identified 110 (67%) that have not previously been reported as PDGF-regulated sites (PhosphoSitePlus)^30^ and are therefore considered novel. Within the phosphoproteomics data set we observed that phosphorylation of Ser303 on insulin receptor substrate 2 (IRS2) increased upon PDGF stimulation (Fig. 2). IRS2 is an adaptor protein that binds to the insulin receptor and regulates insulin signalling pathways. PKC isoforms, β and δ, or angiotensin II can induce phosphorylation of Ser303 on IRS2 in endothelial cells leading to inhibition of insulin signalling^31^. This suggests a novel role for PDGF as a negative regulator of insulin signalling, possibly via PKC, known to be activated downstream of PDGF through PLCγ^32, 33^.

After ERK1/2, the next most significant increase in phosphorylation was observed for Ser72 of Ras-related protein Rab7a (Fig. 2). Rab7a is a small GTPase involved in the regulation of endo-lysosomal trafficking^34^. Phosphorylation of Rab7a on Ser72 inactivates the protein, thereby impairing the recruitment to endosomal membranes and delaying the transport of EGFR from early to late endosomes^35^. This suggests that PDGFR activates an as yet unidentified kinase which phosphorylates Ser72 on Rab7a indicating an intersection between two major RTKs, PDGFR and EGFR in the regulation of endocytosis. In addition, we observed increased phosphorylation of Slit-Robo GTPase-activating protein 3 (srGAP3) on Ser858 (Supplementary Table S2), a RhoGAP that functions downstream of the Robo receptor and is involved in the development of the nervous system^36, 37^. This site is phosphorylated by protein kinase A (PKA), promoting GAP activity towards Rac1 and inhibition of actin reorganisation in neurons^38^.

Following stimulation, 45 novel PDGF-regulated phosphosites were observed to be significantly downregulated including Rho-associated protein kinase 2 (ROCK2; Ser1362), Neurofibromin (NF1; Ser2517), CLIP-associated protein 1 (CLASP1; Ser1193), Parva (Ser14), collapsin response mediator protein 2 (CRMP2; Thr514) and collapsin response mediator protein 4 (CRMP4; Thr514) (Figs 2 and 3; Supplementary Table S2). These proteins are mainly involved in regulation of nervous system homeostasis including development, axon formation and growth cone guidance^39–44^, suggesting a common set of PDGF effectors exist in both fibroblasts and the nervous system and demonstrating that, in addition to the autophosphorylation and phosphorylation of proteins downstream of PDGFR, PDGF can also induce the dephosphorylation of key effectors, some of which regulate cell motility.

### Functional analysis of the PDGF-regulated phosphoproteome

**Figure 4:**
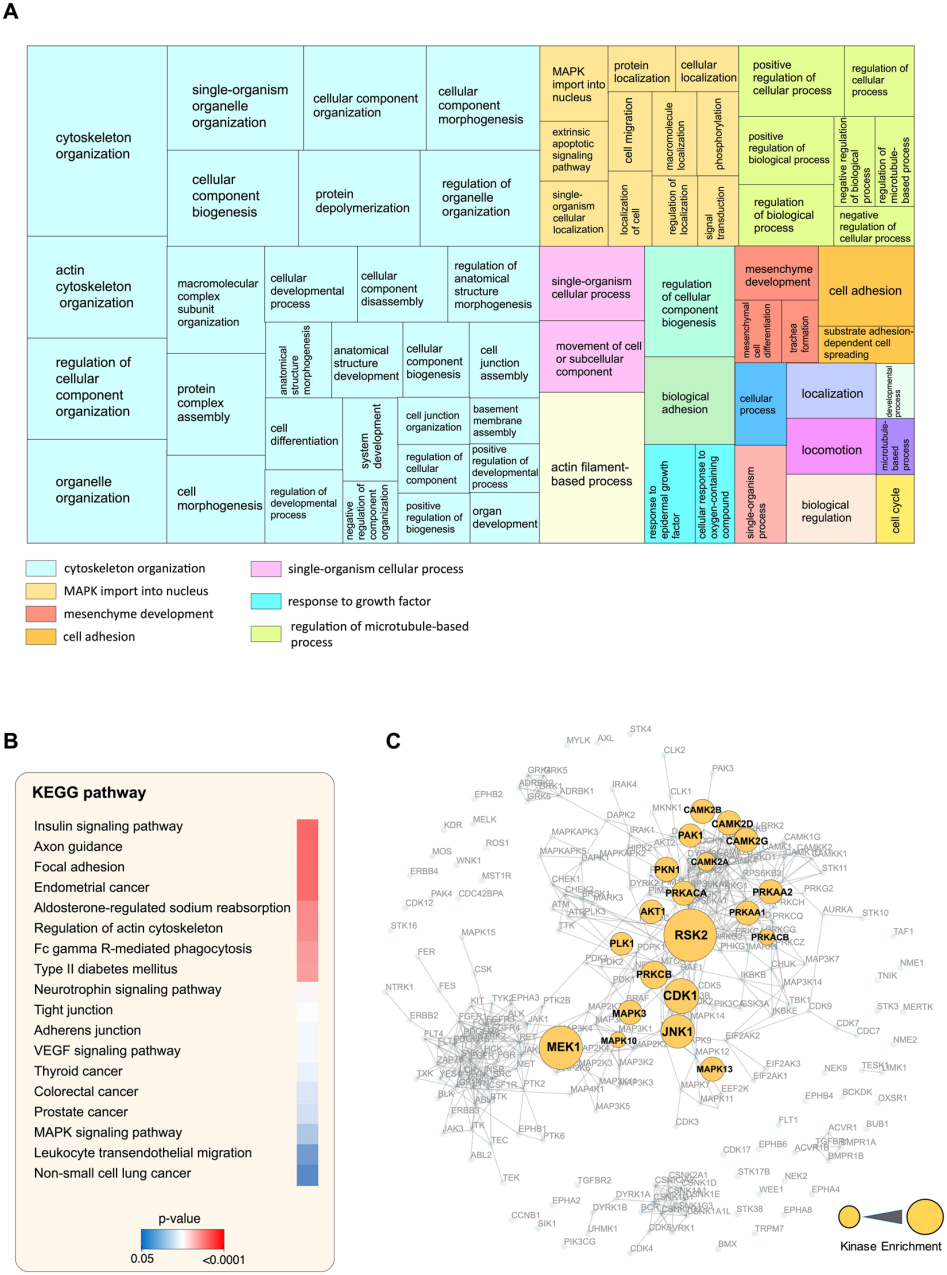
PDGF regulates distinct biological processes and signalling pathways. (**A**) Proteins containing the 161 PDGF regulated phosphosites were analysed in REVIGO to identify enriched GO Terms. Enriched biological processes are visualised as a Tree map. Clusters of related terms are displayed using the same colour and the size of the box corresponds to the −log10 p-value in that category. (**B**) Proteins containing the PDGF-regulated phosphosites were analysed in DAVID to identify enriched KEGG pathways. (**C**) PDGF-regulated phosphopeptides were searched using the Kinase enrichment analysis (KEA) tool. The network shows the enriched predicted upstream kinases and the node size corresponds to the number of PDGF-regulated phosphopeptides predicted to be phosphorylated by that kinase.

Gene Ontology (GO) analysis of the phosphoproteins regulated by PDGF revealed a number of enriched GO biological processes (Fig. 4A; Supplementary Table S3). The dominant enriched GO terms were associated with cytoskeletal organisation, MAP kinase signalling, regulation of microtubules and cell adhesion, all processes involved in cell migration. Actin cytoskeleton and microtubule proteins enriched in our phosphoproteome dataset include LIM domain and actin-binding protein 1 (Lima1), Rho guanine nucleotide exchange factor 17 (Arhgef17), Band 4.1-like protein 1 (Epb41l1) and Stathmin (Stmn1) (Supplementary Table S3). We observed an increase in abundance of phosphopeptides belonging to these proteins in response to PDGF stimulation (Supplementary Table S2). We also note the enrichment of biological processes associated with cell adhesion. Reticulon-4 (Rtn4; also known as Nogo), a protein involved in the regulation of neuron development such as neurite growth and axon-axon adhesion^45, 46^ was phosphorylated on Ser105 in response to PDGF stimulation (Supplementary Table S2). Paxillin (PXN), an adaptor protein involved in modulation of focal adhesions and cell adhesion pathways, is one of the PDGF-regulated proteins^47, 48^. PDGF is known to regulate PXN activity by promoting the phosphorylation of Tyr118^49–51^. We have not identified this site in our phosphoproteomics dataset, but did identify an increase in phosphorylation of Ser83 (Supplementary Table S2). It has been reported that p38MAPK and ERK1/2 can phosphorylate PXN at Ser83 to regulate neurite extension and chemotaxis in neurons^52, 53^.

KEGG pathway analysis of PDGF-regulated proteins revealed enrichment of signalling pathways implicated in cell migration including focal adhesion, regulation of actin cytoskeleton, adherens junctions and transendothelial cell migration (Fig. 4B; Supplementary Table S4)^54–56^. In addition, KEGG analysis of the dataset also identified neurotrophin signalling and axon guidance as PDGF-regulated processes (Fig. 4B). To identify which kinases are activated upon PDGF stimulation, we used the Kinase Enrichment Analysis (KEA) tool^57^. KEA is a web-based software tool for the prediction of kinase-substrate interactions and links mammalian proteins with the upstream kinases that potentially phosphorylate them. KEA analysis revealed a significant enrichment of substrates for ribosomal protein S6 kinase (RSK2) (Fig. 4C; Supplementary Table S5). RSK2 promotes cell motility and invasion by mediating the activity of integrins and actin cytoskeleton rearrangement^58–61^. RSK2 can also promote migration in epithelial cells through the FGFR2-p38 kinase axis pathway^62^. KEA analysis also identified other over-represented kinases including dual specificity mitogen-activated protein kinase kinase (MEK1) and c-Jun N-terminal kinase (JNK1) (Fig. 4C). The activities of both kinases are known to be regulated by growth factors such as PDGF and they are involved in regulation of many cellular functions including cell migration^63, 64^.

### CRMP2 is a novel PDGF-regulated protein

**Figure 5:**
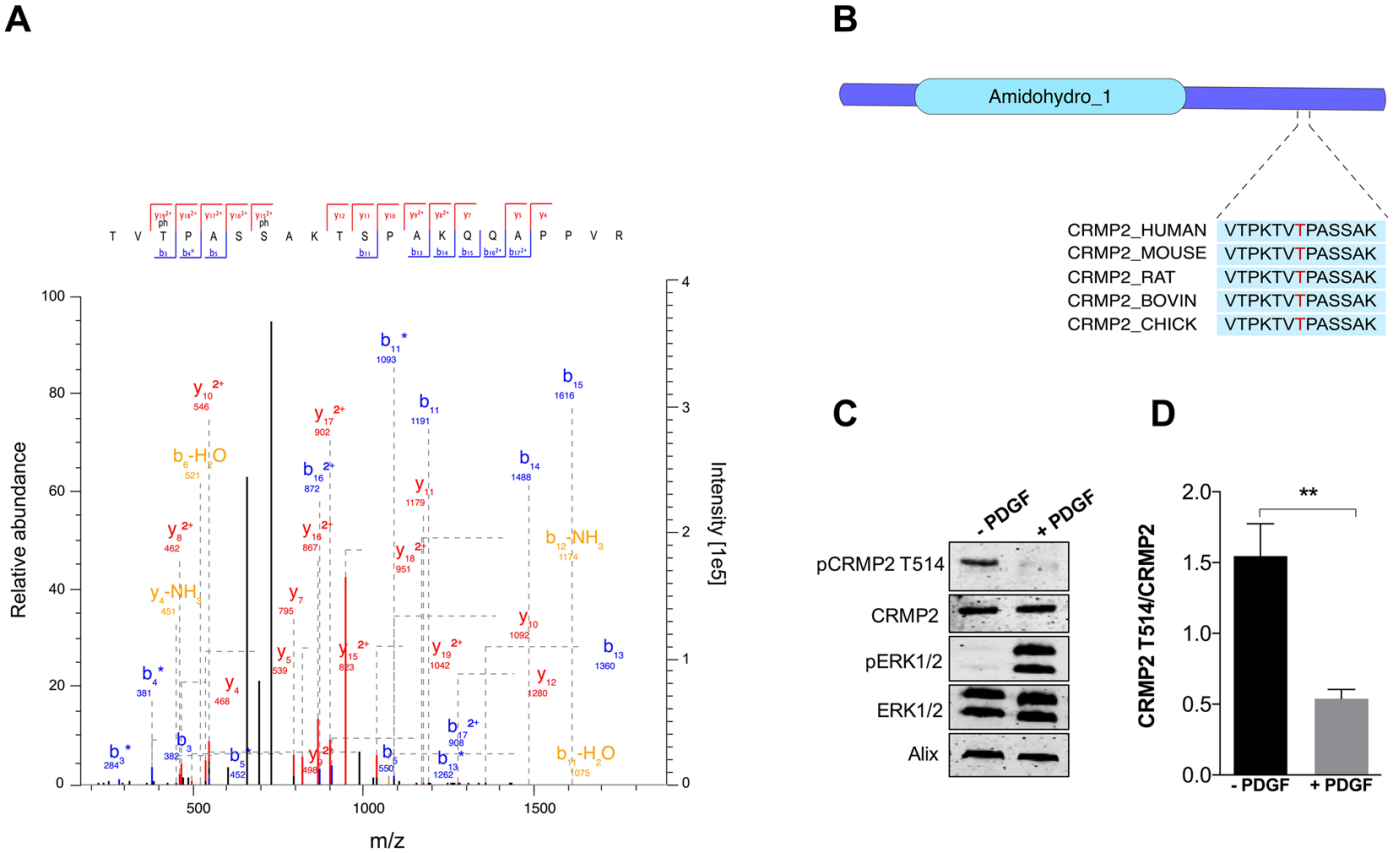
CRMP2 is a novel PDGF-regulated protein. (**A**) Tandem mass spectrum of the CRMP2 T514/S518 phosphopeptide. (**B**) Schematic representation of the domain structure of CRMP2 and the highly conserved amino acid motif containing Thr514. (**C**) MEFs were stimulated with 20 ng/mL PDGF for 7 min. CRMP2, CRMP2 Thr514, ERK1/2, ERK 1/2 Thr202/Tyr204 and Alix in whole cell lysates were analysed by Western blotting. For uncropped images of blots see Supplementary Fig. S2. (**D**) Western blots were quantified and ratios of pCRMP2 to CRMP2 were plotted (**P < 0.01).

﻿We elected to further analyse the role of collapsin response mediator protein 2 (CRMP2) as an example of a novel PDGF-induced dephosphorylation target found within our dataset.﻿﻿ CRMP2 regulates cytoskeletal remodelling, neurotransmission and axon guidance via its ability to promote microtubule assembly^65–67^. Consistent with this, GSK3β has been shown to phosphorylate CRMP2 at Thr509, Thr514 and Ser518 thereby inhibiting CRMP2 activity leading to microtubule depolymerization and destabilization^68, 69^. Within our dataset a doubly phosphorylated peptide from CRMP2 (pThr514; pSer518) was significantly downregulated following PDGF stimulation (Fig. 5A; Supplementary Table S2). Western blotting with a phosphospecific antibody recognising the highly conserved Thr514 residue confirmed that phosphorylation of CRMP2 at Thr514 was significantly decreased following PDGF stimulation compared to non-stimulated cells (Fig. 5B–D). These data clearly show that PDGF regulates the dephosphorylation of CRMP2 at Thr514, an event known to activate the protein.

**Figure 6:**
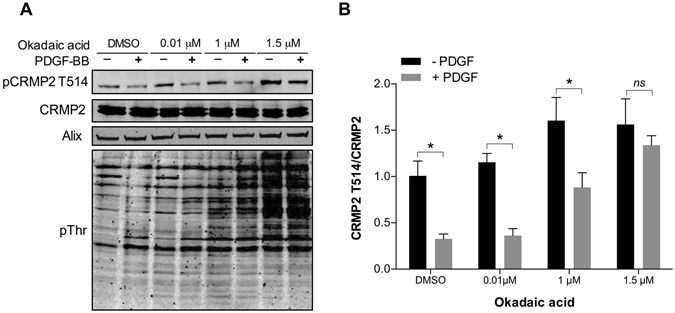
PP1 dephosphorylates CRMP2 Thr514 in response to PDGF stimulation. (**A**) MEFs were incubated with either DMSO or okadaic acid (0.01 μM, 1 μM or 1.5 μM) for 30 min prior to stimulation with 20 ng/mL PDGF for 7 min. CRMP2, CRMP2 Thr514, total phospho-threonine and Alix were analysed by Western blotting. For uncropped images of blots see Supplementary Fig. S3. (**B**) Western blots were quantified and ratios of pCRMP2 to CRMP2 were plotted (*P < 0.05).

It has been previously shown that PP1 or PP2A can dephosphorylate CRMP2 at Ser518, Thr514 or Thr509^70, 71^. We therefore tested the effect of okadaic acid on CRMP2 phosphorylation at Thr514 following PDGF stimulation in MEFs. PP2A is inhibited at low concentrations (<1 μM) of okadaic acid, whereas higher concentrations (>1 μM) inhibit PP1^72, 73^. Western blot analysis showed no significant changes in PDGF-regulated CRMP2 d﻿ephosphorylation when okadaic acid was added at low (0.01 μM or 1 μM) concentrations (Fig. 6A). However, higher concentrations of okadaic acid (1.5 μM) inhibited the ﻿dephosphorylation of CRMP2 at Thr514 in response to PDGF stimulation (Fig. 6A,B). Together, these observations suggest that PDGF signalling acts via PP1 to dephosphorylate CRMP2 at Thr514.

### CRMP2 depletion inhibits cell migration in MEFs

**Figure 7:**
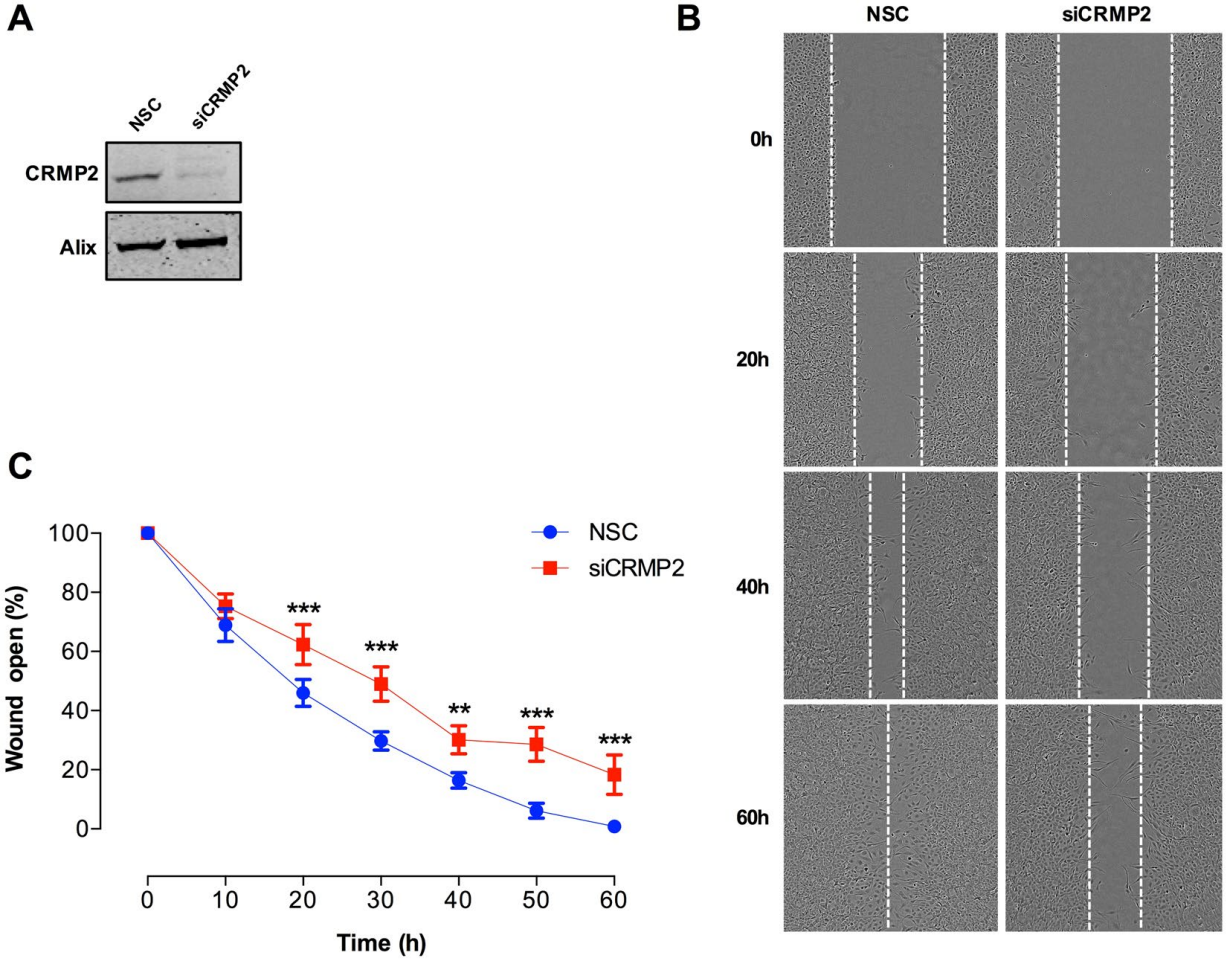
CRMP2 plays a role in PDGF-induced cell migration. (**A**) Representative western blot showing levels of CRMP2 in MEFs following transient transfection with non-silencing control siRNA (NSC), or with CRMP2 siRNA (siCRMP2). Alix was used as a loading control. For uncropped images of blots see Supplementary Fig. S4. (**B**) Transfected MEFs were wounded using an automated scratch maker and stimulated with 20 ng/ml PDGF. Images of five separate wounds were taken at 0, 10, 20, 30, 40, 50, and 60 h post wounding using the IncuCyte ZOOM imaging system. (**C**) The percentage of the wound open at each time point was quantified using ImageJ. Data are presented as average % wound open and error bars represent the standard error of the mean (n = 3; **P < 0.01, ***P < 0.001).

CRMP2 has been widely studied in the nervous system where it regulates neuronal migration and axon guidance^74, 75^. CRMP2 also regulates polarisation and migration of T lymphocytes within the immune system^76, 77^. However, the regulation of cell migration by CRMP2 outside the nervous and immune systems has not been studied. PDGF induced cell migration has been widely studied in fibroblasts, revealing roles for Rac1, MAPK/ERK and JNK kinases^64, 78^. To establish if CRMP2 has a role in PDGF induced cell migration in MEFs we examined the consequences of RNAi-mediated depletion of CRMP2 in a standard *in vitro* scratch wound healing assay. Confluent MEFs were transfected with a CRMP2 directed SMARTpool of siRNAs and non-silencing control siRNA, serum starved and wounded in the presence of PDGF (Fig. 7). The results of this experiment clearly show that siRNA mediated depletion of CRMP2 (Fig. 7A) significantly inhibits the rate of wound closure in the presence of PDGF (Fig. 7B,C). This was confirmed using three individual siRNAs against CRMP2 (Supplementary Fig. S1). These observations confirm that CRMP2, identified in our dataset as a novel PDGF downstream effector, is required for PDGF-stimulated cell migration in MEFs.

## Discussion

PDGF ligands and their cognate receptors are firmly established as key mediators of mesenchymal cell migration in developmental, homeostatic and pathological processes^2–5^. However, the downstream effectors of the PDGF pathway are incompletely characterised. In order to identify PDGF-regulated protein phosphorylation events and expand the range of PDGF mediators, we undertook a study of PDGF-stimulated MEFs using a differential phosphoproteomics approach^18^. The aim of this study was to significantly expand the repertoire of PDGF pathway effectors and to identify novel candidates that might mediate PDGF induced cell migration. Here we report the identification of 989 phosphosites from 611 proteins in our screen. Out of these, 116 phosphopeptides were significantly upregulated in PDGF-stimulated cells, many of which are novel PDGF-regulated phosphosites. Phosphoproteins regulated by PDGF play a role in cellular signalling pathways that mediate cell adhesion, endocytosis and vesicular trafficking, cytoskeletal organization and nuclear function.

We also report the identification of 45 phosphopeptides that are significantly down-regulated in response to PDGF stimulation. These include proteins such as CLASP1 and CRMP2. CRMP2 is of interest as an effector of PDGF-mediated cell migration as it has a well- established role as mediator of axon guidance, growth cone collapse and cell migration in neurons^67, 75, 79^. Phosphoproteomic and western blotting analysis indicated that, in MEFs, PDGF mediates dephosphorylation of CRMP2 at Thr514. In neuronal cells this site is phosphorylated by GSK3β leading to destabilization of microtubules and neurite degeneration, whereas the dephosphorylated form of CRMP2 Thr514 promotes microtubule stabilisation and sustains neurite outgrowth^68, 69, 80^. This indicates that dephosphorylation of CRMP2 involves the activation of a protein phosphatase(s). We showed that micromolar concentrations of okadaic acid, a serine/threonine phosphatase inhibitor, restored the phosphorylation of CRMP2 at Thr514. This is most likely due to preferential inhibition of protein phosphatase 1 (PP1)^72, 73^. CRMP2 is not however dephosphorylated in non-stimulated cells showing that PDGF pathway activation is essential for the CRMP2 directed activity of this phosphatase. Given the intersection of CRMP2 in neuronal cells and MEFs we were interested to learn if CRMP2 was required for PDGF induced cell migration. Depletion of CRMP2 resulted in impairment of cell migration in a scratch wound assay in the presence of PDGF showing that it is required for PDGF induced cell migration in MEFs.

Our quantitative phosphoproteomics study has significantly expanded the known repertoire of PDGF effectors and provided a resource for further experimental studies. The novelty of our findings is highlighted by the fact that we have identified a role for CRMP2 in PDGF-regulated cell migration, a fundamental biological process closely linked to developmental processes and progression of disease.

## Methods

### Phosphoproteomics and bioinformatics

Details of the experimental setup for the quantitative phosphoproteomics experiments have been previously described^18^. Within the MaxQuant output, phosphorylation sites were considered to be localised correctly if the localisation probability was at least 0.75 (75%) and the score difference at least 5. Significance testing was performed in the Perseus software environment, which is part of MaxQuant (Perseus version 1.5.0.15; www.perseus.framework.org), using a Student’s t-test on log 2 transformed ratios and controlled with a Benjamini-Hochberg FDR threshold of 0.05. Peptides quantified in three or more experimental repeats were deemed significantly changed and regulated by PDGF if they had a p-value of <0.05 and a ratio of <0.667 or >1.5 (at least a 1.5-fold change in abundance). The COMPARTMENTS database^81^ was used to assign proteins to subcellular localisations. Protein network visualization was performed using Cytoscape (version 3.3.0)^82^. DAVID (Database for Annotation, Visualization and Integrated Discovery)^83^ was used to identify over-represented KEGG pathways^54–56^ (threshold count, 2; EASE score, 0.05) and REVIGO^84^ was used for identification and visualisation of over-represented GO terms. Kinases upstream of the identified PDGF-regulated phosphopeptides were predicted using Kinase Enrichment Analysis (KEA)^57^.

### Reagents and antibodies

Antibodies against CRMP2 (#9393), CRMP2 Thr514 (#9397), and phospho-threonine (#9381) were purchased from Cell Signaling Technology (Danvers, MA). Antibodies against ERK1/2 (sc-514302) and ERK Thr202/Tyr204 (sc-136521) were purchased from Santa Cruz Biotechnology (Dallas, TX), and against tubulin (T6199) from Sigma-Aldrich Company Ltd (U.K.). The anti-Alix antibody was a gift from Carl Hendrik Heldin (Ludwig Institute for Cancer Research, Uppsala, Sweden)^85^. Goat anti-mouse-IgG IRDye-conjugated antibody (925-68070) and goat anti-rabbit-IgG (925-68071) HRP-conjugated antibodies were from LI-COR Biosciences (Lincoln, NE). Recombinant human PDGF-BB (#8912) was purchased from Cell Signaling Technology and okadaic acid was purchased from Calbiochem (Hertfordshire, UK).

### Cell culture

Mouse embryonic fibroblasts (MEFs) were grown in Dulbecco’s modified Eagle’s medium (DMEM) supplemented with 10% fetal bovine serum (FBS), 100 U/mL penicillin, 10 mg/mL streptomycin, and 250 μg/mL amphotericin B. For growth factor stimulation experiments, cells were grown to 80% confluence and starved for 16 h in serum-free DMEM before being treated with 20 ng/mL recombinant PDGF-BB at 37 °C. In some experiments, cells were incubated with either 0.01 μM, 1 μM or 1.5 μM okadaic acid.

### Cell lysis and immunoblotting

Cell lysates were prepared, separated by SDS-PAGE, transferred to nitrocellulose membranes and immunoblotted using previously described protocols^18^. Immunoblots were visualised using fluorescence detection on the Odyssey Infrared Imaging System (LI-COR Biosciences, Lincoln NE). Densitometric analysis was carried out using ImageJ^86^.

### RNA interference

Cells were transiently transfected with 80 pmol of SMARTpool or individual ONTARGETplus siRNA for murine CRMP2 (L-041965-01-0005, J-041965-09-002, J-041965-10-002, J-041965-11-002; Dharmacon, Lafayette, CO) using Lipofectamine RNAiMAX (Life Technologies, Paisley, UK) as described elsewhere^87^. Nonsilencing control (NSC) siRNA was used as a control for each experiment.

### Scratch wound assay

Cells were plated in 96-well cell culture plates and cultured to confluency. Cells were serum starved overnight. Scratch wounds were made using an automated wound maker (Essen BioScience, Hertfordshire, UK), cells were stimulated with 20 ng/ml PDGF, and images collected at hourly intervals post-wounding using an IncuCyte ZOOM imaging system (Essen BioScience, Hertfordshire, UK).

### Data Availability

The mass spectrometry proteomics data, including the output from MaxQuant^88^, used in this analysis have been previously deposited on the Proteome Xchange Consortium via the PRIDE partner repository with the dataset identifier PXD002545^18, 21^.

## Electronic supplementary material

Supplementary Information

Supplementary Table 1

Supplementary Table 2

Supplementary Table 3

Supplementary Table 4

Supplementary Table 5
