# Supplementary material for: Quantitative Phosphoproteomics Reveals a Role for Collapsin Response Mediator Protein 2 in PDGF-Induced Cell Migration
Source: Sci Rep. 2017 Jun 21;7:3970. doi: 10.1038/s41598-017-04015-x (PMC5479788; doi:10.1038/s41598-017-04015-x)
Supplement: Supplementary file 1 — Supplementary Information [file 41598_2017_4015_MOESM1_ESM.pdf]

Quantitative Phosphoproteomics Reveals a Role for Collapsin Response Mediator Protein 2 in PDGF-Induced Cell Migration

Adil R. Sarhan, Justyna Szyroka, Shabana Begum, Michael G. Tomlinson, Neil A. Hotchin, John K. Heath and Debbie L. Cunningham

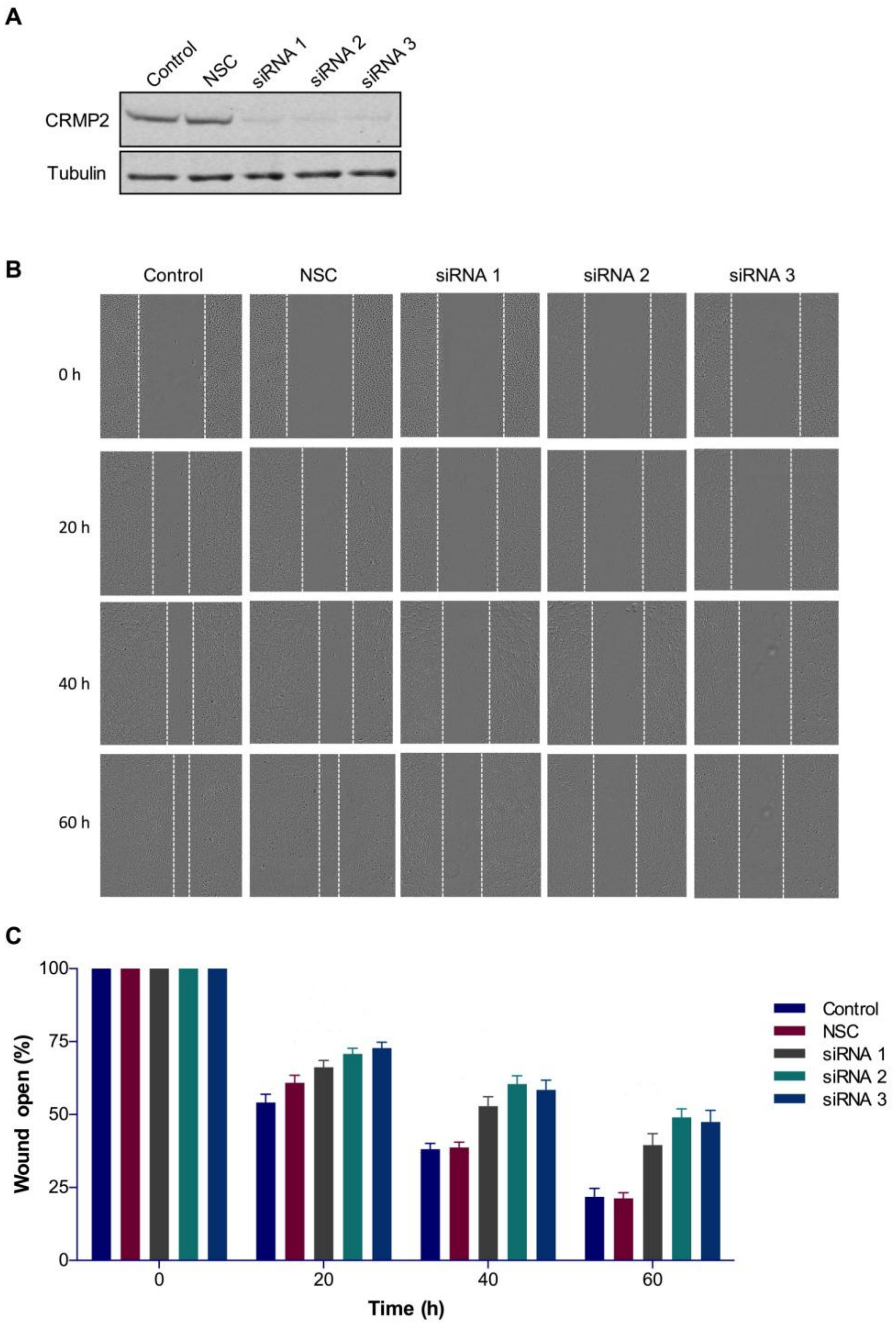

Supplementary Figure S1. PDGF-induced cell migration through CRMP2. (**A**) Western blot showing levels of CRMP2 in MEFs following transient transfection with transfection reagents only (Control), non-silencing control siRNA (NSC), or with three individual CRMP2 siRNAs (siRNA1-3). Tubulin was used as a loading control. (**B**) Transfected MEFs were wounded using an automated scratch maker and stimulated with 20ng/ml PDGF. Images of at least six separate wounds were taken at 0, 20, 40, and 60 h using the IncuCyte ZOOM imaging system (**C**) The percentage of the wound open at each time point was quantified using ImageJ. Data are presented as average % wound open and error bars represent the standard deviation.

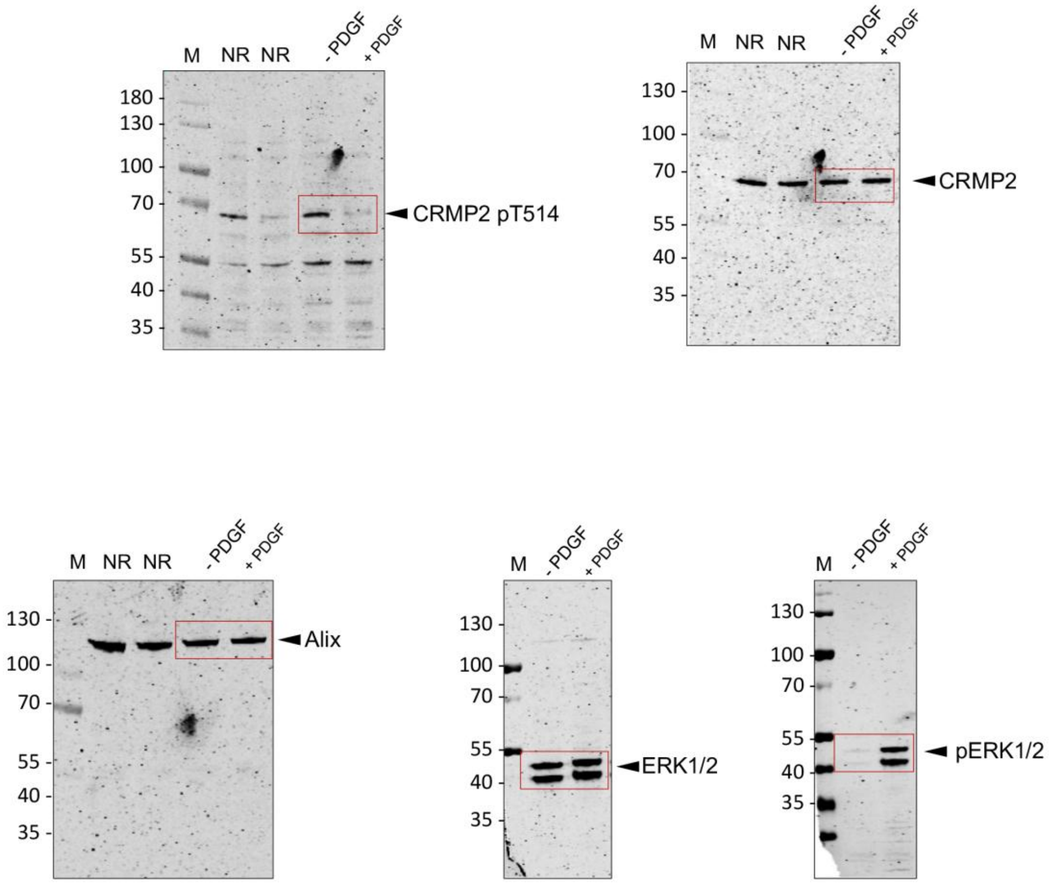

Supplementary Figure S2. MEFs were stimulated with 20 ng/mL PDGF for 7 min. CRMP2, CRMP2 Thr514, ERK1/2, ERK 1/2 Thr202/Tyr204 and Alix in whole cell lysates were analysed by Western blotting. Boxes indicate cropped regions displayed in Figure 5C. M, molecular weight marker; NR, non-relevant sample.

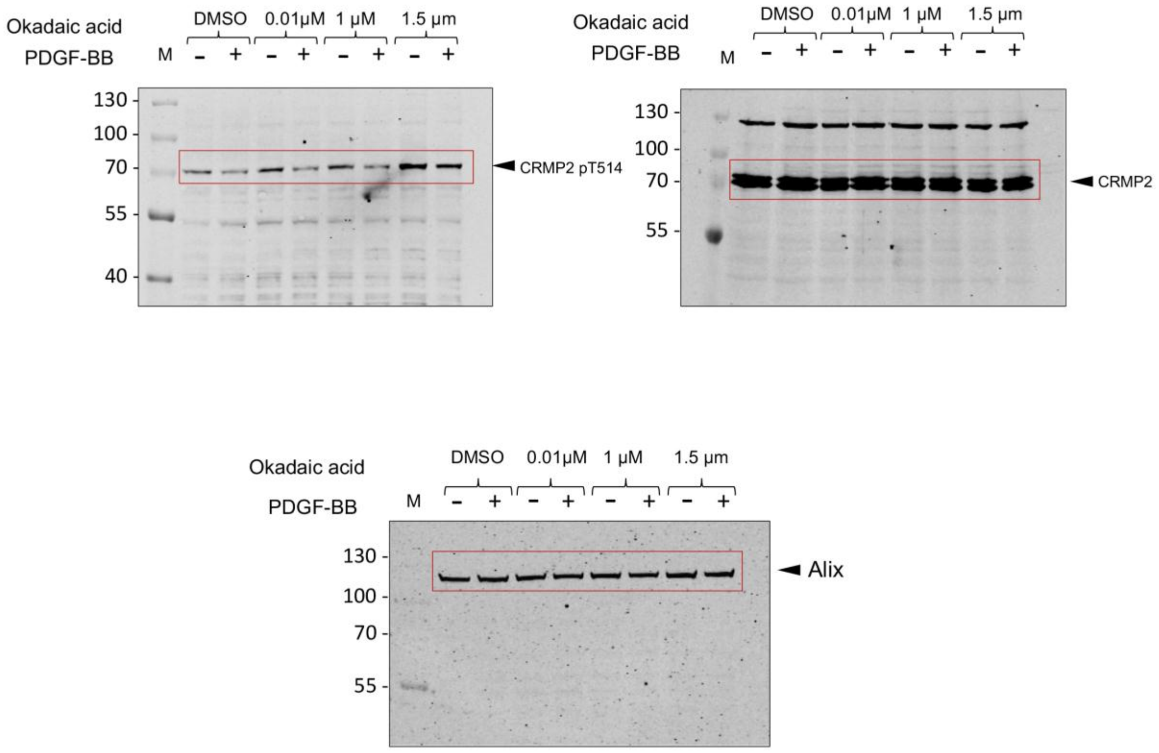

Supplementary Figure S3. MEFs were incubated with either DMSO or okadaic acid (0.01 µM, 1 µM or 1.5 µM) for 30 min prior stimulation with 20ng/mL PDGF for 7 min. CRMP2, CRMP2 Thr514, and Alix were analysed by Western blotting. Boxes indicate cropped regions displayed in Figure 6A. M, molecular weight marker.

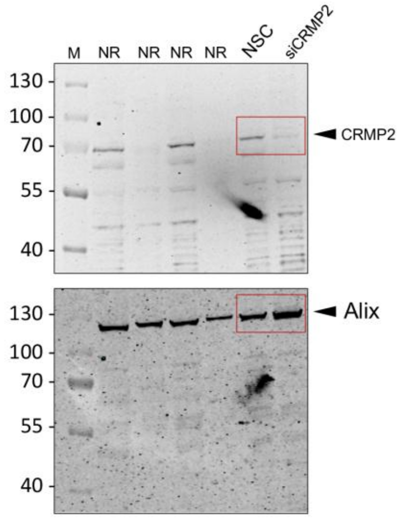

Supplementary Figure S4. Western blot showing levels of CRMP2 in MEFs following transient transfection with non-silencing control siRNA (NSC), or with CRMP2 siRNA (siCRMP2). Alix was used as a loading control. Boxes indicate cropped regions displayed in Figure 7A. M, molecular weight marker; NR, non-relevant sample.
